# Serum levels of periostin and exercise-induced bronchoconstriction in asthmatic children

**DOI:** 10.1016/j.waojou.2018.11.004

**Published:** 2019-01-26

**Authors:** Ju Hwan Cho, Kyubo Kim, Jung Won Yoon, Sun Hee Choi, Youn Ho Sheen, ManYong Han, Junya Ono, Kenji Izuhara, Heysung Baek

**Affiliations:** a Comprehensive Cancer Center, Radiation Oncology, The Ohio State University, Columbus, OH, USA; b Department of Otorhinolaryngology-Head and Neck Surgery, Hallym University Kangdong Sacred Heart Hospital, Republic of Korea; c Department of Pediatrics, Myongji Hospital, Goyang, Republic of Korea; d Department of Pediatrics, Kyung Hee University School of Medicine, Seoul, Republic of Korea; e Department of Pediatrics, CHA University School of Medicine, Seoul, Republic of Korea; f Department of Pediatrics, CHA University School of Medicine, Seongnam, Republic of Korea; g The Shino-Test Corporation, Sagamihara, Japan; h Department of Biomolecular Sciences, Saga Medical School, Saga, Japan; i Department of Pediatrics, Kangdong Sacred Heart Hospital, Hallym University College of Medicine, Seoul, Republic of Korea

## Abstract

**Background:**

Periostin is induced by IL-13 and has been studied as a biomarker of asthma. The present study explored the relationship between serum levels of periostin and exercise-induced bronchoconstriction (EIB) in asthmatic children.

**Methods:**

The study population consisted of 86 children 6–15 years old divided into an asthmatic group (*n* = 56) and healthy controls (*n* = 30). We measured the levels of periostin in serum and performed pulmonary function tests including baseline measurements, post-bronchodilator inhalation tests, exercise bronchial provocation tests (BPTs), and mannitol BPTs.

**Results:**

The 56 asthmatic children were divided into four groups: asthmatics with positive exercise BPT and positive mannitol BPT (*n* = 30), asthmatics with positive exercise BPT but negative mannitol BPT (*n* = 7), asthmatics with negative exercise BPT but positive mannitol BPT (*n* = 10), and asthmatics with negative exercise BPT and negative mannitol BPT (*n* = 9). Serum levels of periostin in asthmatic children with both positive exercise and mannitol BPT were significantly greater than those in asthmatic children with both negative exercise and mannitol BPT (95.0 [75.0–104.0] vs. 79.0 [68.0–82.5] ng/mL, *P* = 0.008) and controls (74.0 [69.75–80.0] ng/mL, *P* < 0.001). Periostin levels were significantly correlated with both the maximum decrease in %FEV_1_ and mannitol PD_15_ value.

**Conclusion:**

Serum levels of periostin in asthmatic children with both positive exercise and mannitol BPT were significantly greater than those in asthmatic children with both negative exercise and mannitol BPT and also greater than in healthy controls.

## Background

Exercise-induced bronchoconstriction (EIB) is an acute phenomenon where the airways narrow as a result of physical exertion. Although EIB is not observed in all cases of asthma, a significant number of asthmatic patients experience exercise-induced respiratory symptoms, as exercise is one of the most common triggers of bronchoconstriction in these patients.^1^ EIB can only be diagnosed when there are changes in lung function induced by exercise, regardless of symptoms.^1^, ^2^ However, the exercise provocation test needed for diagnosis may be difficult for some patients, particularly young children. The development of several possible surrogates for exercise testing, such as eucapnic voluntary hyperpnea or hyperventilation and dry powder mannitol, has facilitated easier diagnosis of EIB.^1^, ^2^

The pathophysiology of EIB has been elucidated over the last two decades.^2^ It is clear that during EIB, inflammatory mediators, including histamine, tryptase, and leukotrienes, are released into the airways from cellular sources, including eosinophils and mast cells.^3^, ^4^ Serum levels of periostin is a promising biomarker of T_H_2-induced airway inflammation,^5^ eosinophilic airway inflammation,^6^ and response to T_H_2-targeted therapy.^5^ Periostin is induced by IL-13, which is a member of the T_H_2 cytokine family and a product of eosinophils, basophils, activated T cells, macrophages, and mast cells.^7^ Periostin is induced by IL-13 and can induce proinflammatory cytokines, including thymic stromal lymphopoietin (TSLP).^8^, ^9^ Recently, it was suggested that TSLP in combination with IL-33 increases mast cell formation of eicosanoids, which are important in patients with EIB.^10^

We hypothesized that periostin levels would be higher in asthmatics with EIB than in healthy children. Furthermore, as we reported previously that serum levels of periostin are associated with airway hyperresponsiveness (AHR) to methacholine and mannitol,^11^ we also hypothesized that periostin may be correlated with AHR induced by exercise in asthmatic children. Our objective was to evaluate the relationship between serum levels of periostin and EIB in asthmatic children.

## Methods

### Subjects

Subjects were recruited from outpatient clinics at Hallym University Kangdong Sacred Heart Hospital, Seoul, Korea. We systematically recruited all new asthma patients at their first visits because of suspected asthma, and all diagnoses were verified by clinical examination, pulmonary function testing, and methacholine bronchial provocation tests (BPTs). The patients were newly diagnosed with asthma and had undergone maintenance therapy for 0.5–2 years. Asthma was defined as the presence of symptoms with less than 16.0 mg/mL inhaled methacholine, which induced a 20% decrease in FEV_1_ (PC_20_).^12^ Severity of asthma was classified according to the guidelines of the Global Initiative for Asthma (GINA) using an algorithm including medication dose, FEV_1_, medication adherence, and symptom levels.^13^ Patients were given inhaled short-acting β_2_-agonists on demand to relieve symptoms, with or without controller medications (inhaled corticosteroids, leukotriene receptor modifier, or long-acting β_2_-agonists). The controls were healthy children matched by age and gender, who had applied for a routine health checkup or vaccination. They had no history of wheezing or infection over the 2 weeks before the study. Exclusion criteria included acute exacerbation of asthma requiring systemic corticosteroids during the previous 6 months and parenchymal lung disease evident in chest radiographs performed 4 weeks before the study. Of the healthy controls, those at any risk for atopy or subclinical eosinophilic inflammation were excluded using fractional exhaled nitric oxide (FeNO) of ≥20 parts per billion (ppb).^14^ Atopy was defined as the presence of at least one positive allergen-specific IgE test result (IgE ≥ 0.35 kU/L) or a positive finding in skin prick tests (SPTs).

### Study protocol

**Fig. 1:**
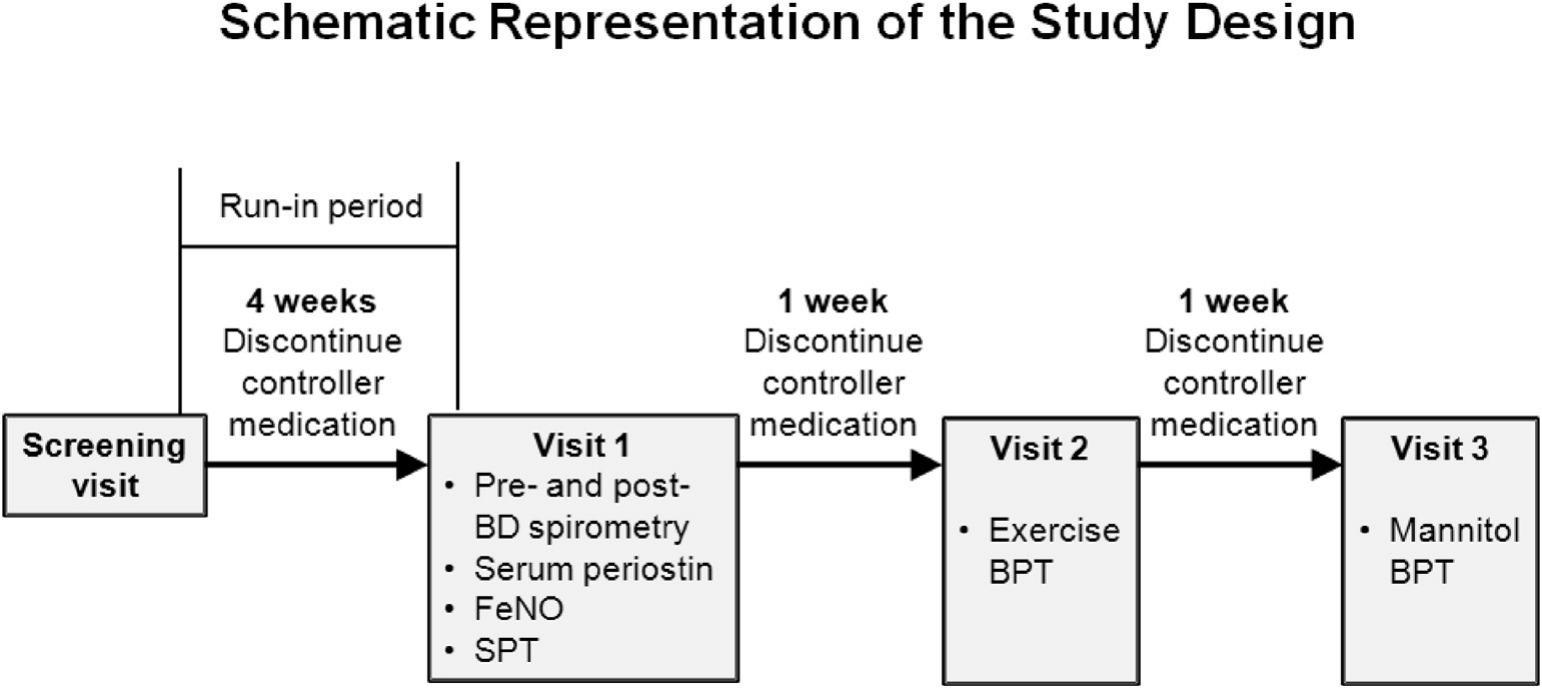
Study schema.

A schema of the study design is shown in Fig. 1. After a 4-week run-in period, the asthmatic patients made three visits to our clinic at the same time of day. During the observation period, all patients were asked to discontinue controller medications, and were excluded if they experienced asthma exacerbations requiring recommencement of such medication. At the first of the three visits, blood samples were taken and FeNO levels were measured by a physician. Each subject was evaluated using SPTs and pre- and post-bronchodilator spirometry. At the second and third visits, separated by intervals of at least 1 week, BPTs with exercise and mannitol challenge were performed. Healthy controls made two visits to our clinic at the same time of day. At the first of the three visits, FeNO levels were measured by a physician and each subject was evaluated using SPTs. Those at no risk for atopy or eosinophilic inflammation were included and made a second visit. At the second visit, blood samples were taken and exercise challenges were performed. Blood samples were stored at −70 °C before determining the periostin levels in serum. At the third visit, mannitol challenge was performed. The spirometry and exercise challenge tests were performed by a trained technician. All procedures were approved by the Medical Ethics Committee of Hallym University Kangdong Sacred Heart Hospital, Seoul, Korea, and all subjects and/or parents provided written informed consent.

### Exercise challenge

Exercise challenges were conducted in accordance with American Thoracic Society standards^12^ and performed by running on a treadmill with the nose clipped (LE 200 CE; Jaeger Co., Freiburg, Germany) using a standardized protocol. Heart rate was monitored continuously with a radiographic device (electronic ECG monitor, BCI^®^ Autocorr^®^; Smiths Medical PM, Inc., Waukesha, WI). The temperature in the laboratory was maintained at 22 °C, with humidity of 40–50%. Inspired air temperature and humidity were measured. The treadmill speed was increased until the heart rate was ∼85% of the predicted maximum ([220–age] × 0.9) and maintained for 6 min. Spirometry was performed 20 and 5 min before each exercise challenge and repeated 0, 3, 6, 10, 15, and 20 min afterwards. The results of exercise challenges were considered positive with a ≥15% decrease in FEV_1_ after exercise.^12^

### Mannitol BPT

Dry powder mannitol (Aridol; Pharmaxis, French's Forest, NSW, Australia) was administered according to the manufacturer's recommendations, and FEV_1_ values were recorded as prescribed by current guidelines.^15^ The FEV_1_ recorded after inhalation of a placebo capsule served as the baseline value. The challenge was completed when a ≥15% drop in FEV_1_ from baseline occurred, which was considered a positive response, or when the maximum cumulative dose of mannitol (635 mg) was administered. Asthmatics exhibiting a decrease in FEV_1_ of at least 15% from baseline after inhalation of ≤635 mg mannitol were enrolled in the positive-mannitol BPT group, and the others were placed in the negative-mannitol BPT group. For positive challenge results, the cumulative provocative dose causing a 15% drop in FEV_1_ (PD_15_ dose) was calculated by log-linear interpolation of the final two data points. The responses to mannitol were expressed as PD_15_ values.

### FeNO

FeNO was measured using a portable nitric oxide analyzer (NIOX MINO^®^; Aerocrine, Solna, Sweden) that provided measurements at an exhalation flow rate of 50 mL/s expressed in ppb.^16^ Determinations made with the device were in clinically acceptable agreement with measurements provided by a stationary analyzer according to the guidelines of the American Thoracic Society.^17^

### Measurement of serum biomarkers

Blood samples were obtained between 08:00 and 09:00. Periostin levels were measured by Shino Test Corp. (Kanagawa, Japan) using an enzyme-linked immunosorbent assay (ELISA), as described previously.^18^ Briefly, The SS18A mAb (2 μg mL^−1^) was incubated overnight at 25 °C on ELISA plates (Thermo Fisher Scientific, Rochester, NY, USA). Then the ELISA plates were blocked by blocking buffer (0.5% casein in TBS, pH 8.0) overnight at 4 °C and then washed three times with washing buffer (0.05% Tween20 in PBS). To measure periostin levels, diluted (1/100–1/200) serum samples or recombinant periostin standards were added and incubated for 18 h at 25 °C. After washing five times, the peroxidase labeled SS17B mAb (50 ng mL^−1^) was added followed by incubation for 90 min at 25 °C. After washing five times to remove excess Ab, reaction solution (0.8 mM 3,3′,5,5′-Tetramethylbenzidine, 2.5 mM H_2_O_2_) was added, followed by incubation for 10 min at 25 °C and then the reaction was stopped by adding the stop solution (0.7 N HCl). The values were calculated by subtracting the absorbance at 550 from the absorbance at 450 nm. Periostin concentrations in the serum were calculated simultaneously using the recombinant periostin proteins. The assay was performed in duplicate.

### Statistical analyses

The data were analyzed using SPSS ver. 21.0 (SPSS, Chicago, IL). Continuous data are expressed either as means with standard deviations or as medians with interquartile ranges depending on the data distribution. Groups were compared using the Kruskal–Wallis test for continuous variables or χ^2^ tests for categorical variables. *Post hoc* pairwise comparisons were performed using the Tamhane test. Numerical parameters with non-normal distributions (periostin level, maximum percentage change in FEV_1_ from baseline to after exercise and mannitol PD_15_ values) were log-transformed. Correlations between periostin levels, lung function, total IgE levels, eosinophil counts in peripheral blood (PB), eosinophil cationic protein (ECP) levels, and FeNO values were evaluated by calculating Spearman's rho. The effects of log-transformed periostin levels on the log-transformed maximum percentage change in FEV_1_ from baseline to after exercise, and mannitol PD_15_ data were analyzed by linear regression to allow adjustment for age, sex, atopy, and PB eosinophil count. The estimates obtained were regression slopes for log-transformed periostin levels against the log-transformed maximum percentage change in FEV_1_ from baseline to after exercise and mannitol PD_15_ value. The overall test performance of periostin for identifying asthmatic patients with positive exercise BPT and for identifying asthmatic patients with positive mannitol BPT was reviewed based on receiver operating characteristic (ROC) curve analyses. The overall accuracy of the test was measured as the area under the ROC curve (AUC). The prevalence of disease used in the analyses was estimated from the ratio of positive and negative cases in the dataset. The 95% confidence interval (CI) for test characteristics was calculated using MedCalc v.14.8.1 (MedCalc, Mariakerke, Belgium).

## Results

### Characteristics of the study subjects

A total of 90 subjects were recruited and took part in this study. The patient group consisted of 60 asthmatics and a control group included 30 healthy subjects. During the run-in period, four subjects with asthma dropped out because of a failure to discontinue controller medications due to exacerbation of asthma. Eighty-six subjects who finished the study were enrolled in the final analyses (56 asthmatics and 30 controls). The 56 asthmatic children were divided into four groups: asthmatics with positive exercise BPT and positive mannitol BPT (*n* = 30), asthmatics with positive exercise BPT but negative mannitol BPT (*n* = 7), asthmatics with negative exercise BPT but positive mannitol BPT (*n* = 10), and asthmatics with negative exercise BPT and negative mannitol BPT (*n* = 9). The demographic data and pulmonary function parameters of the subjects are summarized in Table 1. There were no differences between the asthmatic and healthy children in age, sex, or body mass index (BMI). Of the 56 subjects with asthma, 14 had mild intermittent asthma, 23 had mild persistent asthma, and 19 had moderate asthma according to the GINA guidelines. There were no statistically significant differences in atopy, prior inhaled corticosteroid (ICS) use, or asthma severity among the four asthmatic groups.

**Table 1:** Characteristics of study subjects.

	Exercise (+) Mannitol (+) (n = 30)	Exercise (+) Mannitol (−) (n = 7)	Exercise (−) Mannitol (+) (n = 10)	Exercise (−) Mannitol (−) (n = 9)	Healthy controls (n = 30)	*P* value^a^
Age (y)	8.4 ± 2.2	9.7 ± 2.1	10.6 ± 2.4	9.2 ± 3.0	9.4 ± 3.2	.135
BMI (kg/m^2^)	19.2 ± 3.8	17.8 ± 2.1	18.1 ± 3.4	18.5 ± 4.1	17.7 ± 3.7	.745
Male/female sex						.363^b^
Male, no. (%)	19 (63.3)	5 (71.4)	9 (30.0)	5 (55.6)	15 (50)	
Female, no. (%)	11 (36.7)	2 (28.6)	1 (10.0)	4 (44.4)	15 (50)	
Prior ICS use (%)	16 (53.3)	3 (42.9)	4 (40.0)	3 (33.3)	NA	.706
Asthma severity					NA	.144^b^
Mild intermittent, n (%)	4 (13.3)	2 (28.6)	4 (40.0)	4 (44.4)	NA	
Mild persistent, n (%)	11 (36.7)	4 (57.1)	4 (40.0)	4 (44.4)	NA	
Moderate, n (%)	15 (50.0)	1 (14.3)	2 (20.0)	1 (11.1)	NA	
Atopy (%)	84.2	85.7	80	75	NA	.302
Lung function						
FEV_1_ (pred %)	85.8 ± 17.1^f^	88.4 ± 14.5^f^	81.9 ± 7.8^f^	93.0 ± 15.9^f^	102.2 ± 10.0	<.001
FVC (pred %)	96.8 ± 10.5	92.6 ± 8.8	96.0 ± 13.5	103.4 ± 25.4	98.2 ± 9.7	0.563
FEV_1_/FVC ratio	77.8 ± 12.8^f^	86.4 ± 7.6^f^	81.9 ± 7.8^f^	87.4 ± 10.9^f^	91.1 ± 6.6	<.001
Postbronchodilatory ΔFEV_1_ (pred %)	11.1 ± 15.6^e^^,^^f^	6.1 ± 9.4^f^	9.6 ± 11.9^f^	3.9 ± 5.5	1.2 ± 3.4	.003
Methacholine PC_20_ (mg/mL)	3.3 ± 4.8	2.9 ± 2.4	3.8 ± 3.3	4.2 ± 5.8	NA	.180
Maximum decrease in FEV_1_ after exercise, %	25.1 ± 14.3^c^^,^^d^^,^^e^^,^^f^	18.1 ± 4.5^d^^,^^e^^,^^f^	6.3 ± 1.8^f^	5.3 ± 2.5	3.8 ± 6.1	<.001
Mannitol PD_15_ (mg)	132.0 (83.5–223.2)^d^	NA	321.4 (141.1–419.5)	NA	NA	0.020
Periostin (ng/mL)	95.0 (75.0–104.0)^e^^,^^f^	91.0 (79.0–102.0)	78.0 (58.0–105.0)	79.0 (68.0–82.5)	74.0 (69.75–80.0)	.001
Total IgE (IU/mL)	241.3 (87.7–332.0)^f^	431.8 (366.3–702.2)^f^	511.0 (190.7–2598.6)^c^	176.9 (54.8–552.2)^f^	62.4 (29.3–85.6)	.745
PB eosinophil (/mL)	390.0 (289.5–695.0)^f^	300.0 (92.5–545.0)^c^	420.0 (34.0–532.5)^c^	330.0 (130.1–482.5)^f^	125.0 (95.0–175.0)^f^	.363
FeNO (ppb)	39 (32.8–45.2)^e^^,^^f^	32 (16.0–47.9)^f^	13.8 (8.7–22.8)^f^	16.5 (9.1–23.9)^f^	11.0 (8.0–13.5)^f^	<.001

BMI, body mass index; ICS, inhaled corticosteroid; NA, not applicable; pred %, predicted %; PD_15_, the cumulative provocative dose causing a 15% fall in FEV_1_; PC_20_, the provocative concentration of methacholine inducing a 20% fall in FEV_1_.

Data are presented as absolute numbers (percentages), or as means ± standard deviations, medians (interquartile ranges), depending on their distribution.

a Kruskal-Wallis test.

b Chi-squared test.

c P < 0.05 versus asthmatics with positive exercise BPT and with negative mannitol BPTs (Post hoc pairwise comparisons are Tamhane tests).

d P < 0.05 versus asthmatics with negative exercise BPT and with positive mannitol BPTs (Post hoc pairwise comparisons are Tamhane tests).

e P < 0.05 versus asthmatics with negative exercise BPT and with negative mannitol BPTs (Post hoc pairwise comparisons are Tamhane tests).

f P < 0.05 versus healthy controls (Post hoc pairwise comparisons are Tamhane tests).

### Pulmonary function, AHR to methacholine, exercise, or mannitol

As expected, the baseline FEV_1_ levels and FEV_1_/forced vital capacity (FVC) ratios were significantly lower in asthmatics than in healthy controls, while the bronchodilator responses were significantly greater (Table 1). In group comparisons, the bronchodilator responses were significantly greater in asthmatics with both positive exercise BPT and positive mannitol BPT than in asthmatics with negative exercise BPT and with negative mannitol BPT. There were no differences in methacholine PC_20_ among the four asthmatic groups. The maximum decrease in FEV_1_ after exercise was significantly greater in asthmatics with positive exercise BPT and positive mannitol BPT than in the other asthmatic groups.

### Biomarkers in the peripheral blood of study subjects

**Fig. 2:**
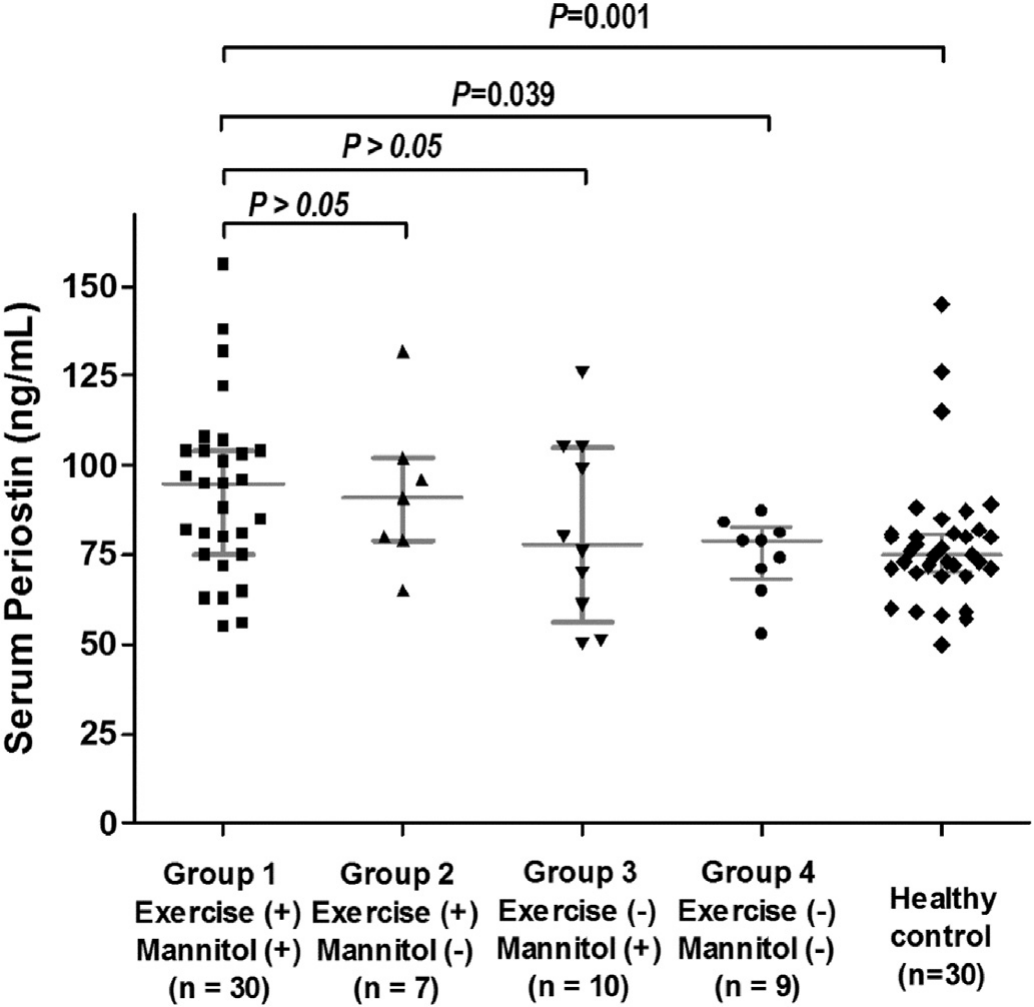
Asthmatics had significantly higher levels of serum periostin than did controls (76.0 (65.0–91.8) vs. 71.0 (57.5–80.0) ng/mL; *P* = 0.017). The horizontal lines in the dot plots represent median and interquartile ranges.

Biomarker levels are shown in Table 1. The total IgE levels, PB eosinophil counts, and FeNO levels were significantly higher in asthmatics than in healthy controls. The total IgE levels and PB eosinophil counts were not significantly different among the four asthma groups. FeNO levels were significantly greater in asthmatic children with positive exercise BPT and positive mannitol BPT than in those with negative exercise BPT and negative mannitol BPT (39 [32.8–45.2] vs. 16.5 [9.1–23.9] ppb; *P* = 0.039) and in controls (vs. 11.0 [8.0–13.5] ppb, *P* < 0.001) (Table 1). Serum levels of periostin were significantly greater in asthmatic children with positive exercise BPT and positive mannitol BPT than in those with negative exercise BPT and negative mannitol BPT (95.0 [75.0–104.0] vs. 79.0 [68.0–82.5] ng/mL, *P* = 0.039) and controls (74.0 [69.75–80.0] ng/mL, *P* = 0.001) (Table 1; Fig. 2).

### Associations between periostin levels and lung function, markers of atopy, AHR to exercise, and AHR to mannitol

Periostin levels were not significantly correlated with lung function but were significantly correlated with PB eosinophil levels (Spearman's correlation coefficient r = 0.331, *P* = 0.015), FeNO (r = 0.384, *P* = 0.004), and total IgE levels (r = 0.384, *P* = 0.004). There were no significant correlations between serum levels of periostin, age, and sex in any group. After adjusting for age, sex, atopy, and PB eosinophil level, the log (periostin level) was significantly associated with the log (maximum decrease in FEV_1_ after exercise) (exhibiting a 0.833% increase for each doubling of the biomarker level from baseline, *P* = 0.010), and with the log (mannitol PD_15_) (2.885% decrease, *P* = 0.009) (Table 2).

**Table 2:** The results of multiple linear regression modeling of changes in maximum decrease in FEV_1_ and mannitol PD_15_ and after exercise in the asthmatic children.

	Log Maximum decrease in FEV_1_ after exercise			Log Mannitol PD_15_		
	Estimate	95% CI	P value	Estimate	95% CI	P value
Gender	0.176	−0.399 to 0.750	0.618	0.246	−0.192 to 0.684	.249
Age	0.027	−0.069 to 0.123	0.575	0.03	−0.056 to 0.116	.466
Atopy	−0.402	−0.813 to 0.010	0.055	−0.28	−1.457 to 0.896	.619
Log periostin level	0.833	0.207 to 1.460	0.010	−2.885	0.968 to 0.511	.009

^∗^P values were computed using a regression model evaluating differences of estimates (slopes) from zero.

### Periostin for differentiating asthmatic patients with positive exercise BPT from those with negative exercise BPT

**Fig. 3:**
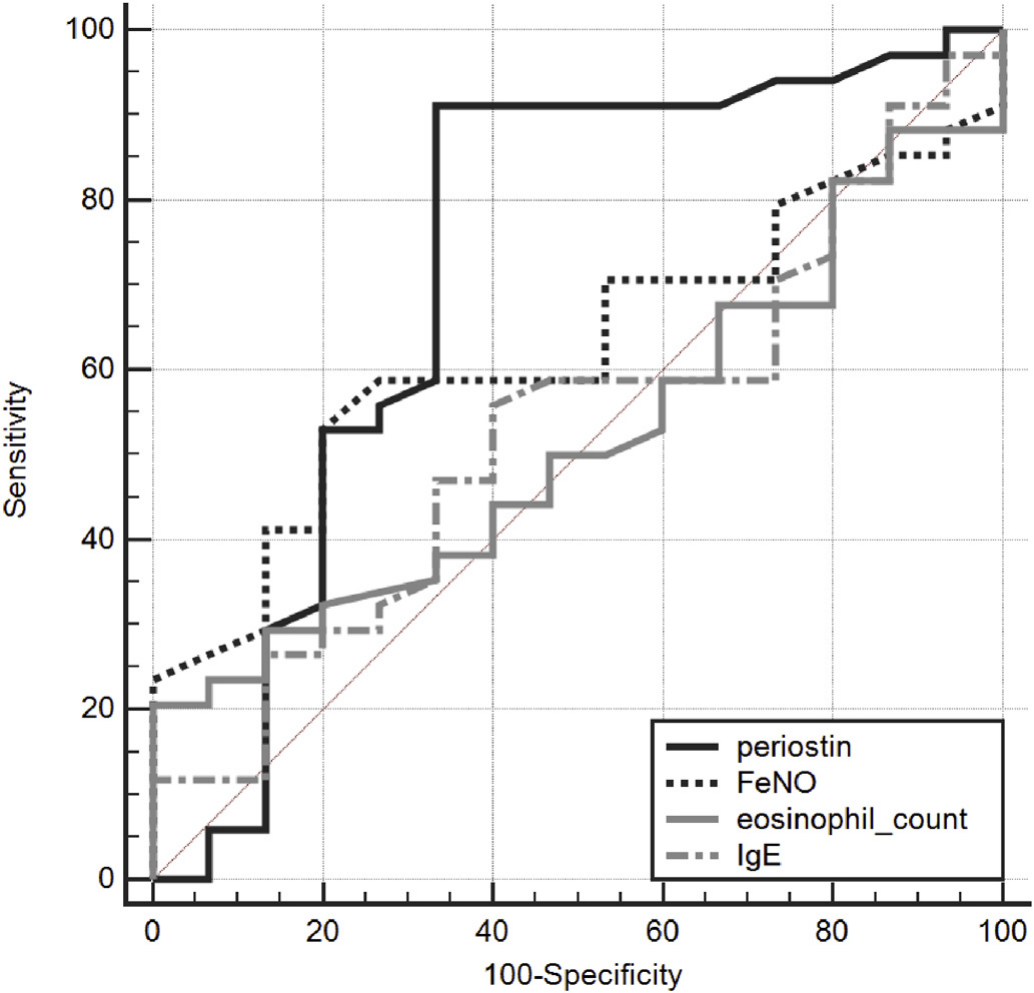
Receiver operating characteristic curve for periostin levels for predicting exercise induced bronchoconstriction.

**Fig. 4:**
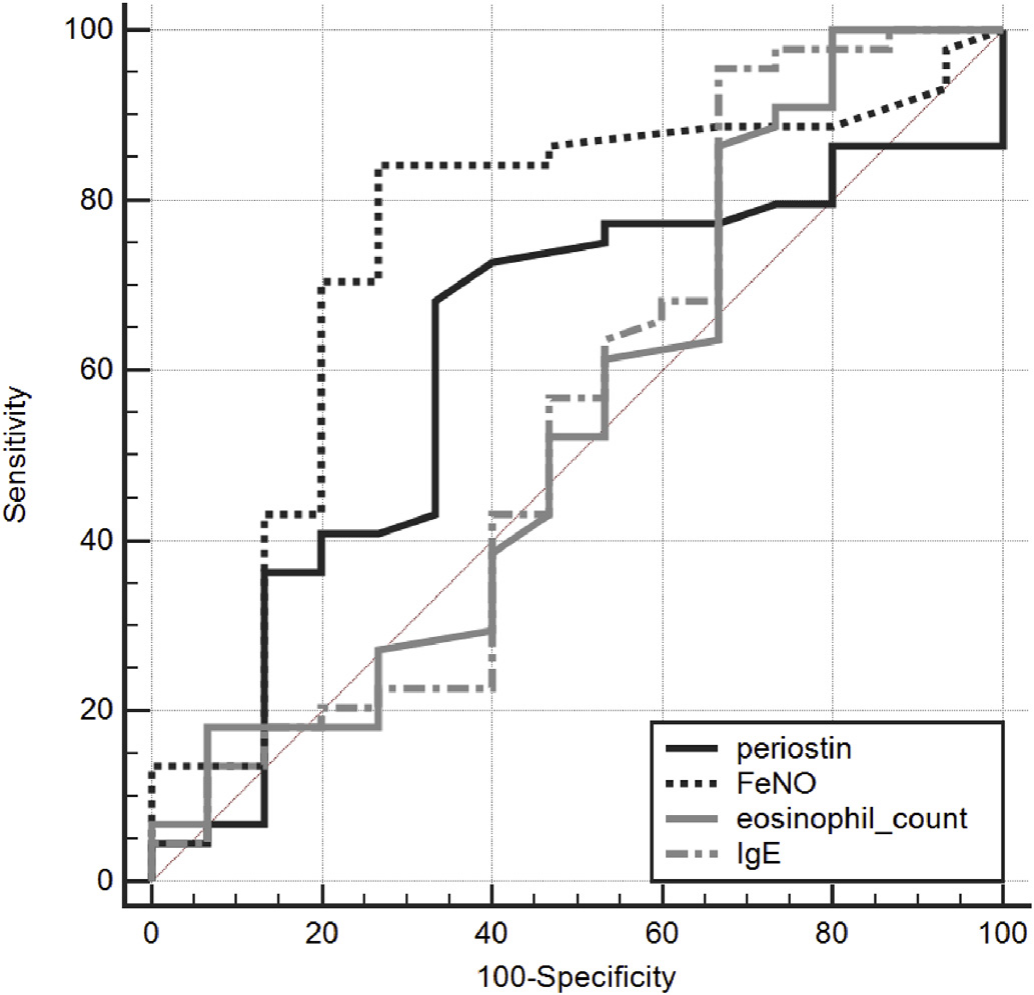
Receiver operating characteristic curve for periostin levels for predicting mannitol-induced bronchoconstriction.

Table 3 shows the ROC curve for using periostin levels to predict positive exercise BPT and to predict positive mannitol BPT. To differentiate asthmatic patients with EIB from those without EIB, the ROC curve for using periostin level had an AUC of 0.722 (Fig. 3). To differentiate asthmatic patients with positive exercise BPT from those with negative exercise BPT, the ROC curve for using FeNO level, eosinophil count, and total IgE had AUCs of 0.625, 0.519, and 0.530, respectively. To discriminate asthmatic patients with positive mannitol BPT from those with negative mannitol BPT, the ROC curve had an AUC of 0.596 (Fig. 4). The AUCs of periostin level, FeNO level, eosinophil count, and total IgE level did not differ significantly. To discriminate asthmatic patients with positive mannitol BPT from those with negative mannitol BPT, the ROC curve for using FeNO level, eosinophil count, and total IgE had AUCs of 0.733, 0.537, and 0.520, respectively. The AUCs of periostin level, FeNO level, eosinophil count, and total IgE level did not differ significantly among the groups.

**Table 3:** The ROC curve analysis of the serum periostin levels for predicting positive exercise BPT and for predicting positive mannitol BPT.

	Biomarkers	AUC	SE	95% CI
For predicting positive exercise BPT	periostin	0.722	0.0949	0.575 to 0.840
	FeNO	0.625	0.0806	0.475 to 0.759
	Eosinophil	0.519	0.0851	0.371 to 0.664
	IgE	0.530	0.0886	0.383 to 0.674
For predicting positive mannitol BPT	periostin	0.596	0.0893	0.459 to 0.723
	FeNO	0.733	0.0855	0.600 to 0.841
	Eosinophil	0.537	0.102	0.401 to 0.669
	IgE	0.520	0.102	0.385 to 0.653

ROC, receiver operating characteristic; AUC, area under curve; SE, standard error; CI, confidence interval.

## Discussion

We investigated the relationship between serum levels of periostin and EIB in pediatric asthma patients. The inflammatory cells most commonly involved in the pathogenesis of EIB are mast cells and eosinophils.^10^, ^19^, ^20^ Mast cells secrete PGD_2_, cysteinyl leukotriene receptor (CysLT), and histamine, which are mediators that trigger airway smooth muscle contraction, sensory nerve activation, and mucus secretion. Mast cells and eosinophils also produce IL-13, a pleiotropic T_H_2 cytokine, which is also secreted by basophils, activated T cells, and macrophages.^7^ Periostin is induced by IL-13, and we showed previously that serum levels of periostin are significantly higher in asthmatic children than in healthy controls.^11^ In this study, serum levels of periostin were significantly greater in asthmatic children with both positive exercise BPT and positive mannitol BPT than in those with both negative exercise BPT and negative mannitol BPT and also healthy controls.

Several studies have shed light on the possible mechanism by which periostin may be involved in EIB. Masuoka et al.^8^ reported that periostin acts directly on keratinocytes via α_v_ integrin to induce secretion of proinflammatory cytokines, including TSLP.^7^ A recent study also showed that periostin is produced by mast cells and can act directly on epithelial cells via integrin-binding activation, resulting in TSLP secretion.^9^ TSLP, in turn, was shown to intensify the EIB-associated granule phenotype and increase IgE receptor-mediated CysLT production in human cord blood-derived mast cells.^10^ Based on these reports, we speculated that periostin may be associated with EIB via TSLP, but further studies are required to clarify this association.

In addition to mast cells, eosinophils seem to play a major role in the pathogenesis of EIB. Peripheral blood eosinophil counts are associated with severity of EIB,^21^ and asthmatic patients with EIB are more likely to have a greater concentration of eosinophils in sputum than those without EIB.^4^, ^22^ In the present study, PB eosinophil counts were significantly higher in asthmatics than in healthy controls, but these levels were not significantly different among the four asthma groups. FeNO is a possible biomarker of airway inflammation in asthma, as it is correlated with eosinophilic activity in the airway. Scollo et al.^23^ reported that the baseline FeNO value was related to the extent of post-exercise bronchoconstriction, suggesting that the FeNO level may predict AHR to exercise. One study showed that FeNO levels were significantly predictive of EIB in atopic wheezy children,^24^ while another demonstrated that FeNO level can be used to screen asthmatic children to determine the need for EIB testing.^25^ In agreement with these observations, we also found that the FeNO levels in asthmatic children with positive exercise BPT and positive mannitol BPT were significantly greater than those in asthmatic children with negative exercise BPT and negative mannitol BPT as well as controls.

Serum levels of periostin, eosinophil counts, and FeNO levels all reflect a T_H_2-driven inflammatory response, but the relationship between these distinct biomarkers may be complex and variable.^26^, ^27^ Jia et al.^28^ collected peripheral blood, sputum, and bronchoscopy biopsy samples to identify noninvasive biomarkers of T_H_2 inflammation in asthmatic patients, and observed that while both FeNO and periostin levels were consistently low in eosinophil-low patients, FeNO showed a greater overlap between eosinophil-low and eosinophil-high subjects. In the present study, periostin levels were significantly correlated with both PB eosinophil and FeNO levels. We found that not only FeNO levels but also periostin were associated with EIB in asthmatic children. After adjusting for age, atopy, and PB eosinophil count, serum levels of periostin were significantly associated with EIB.

EIB is frequently documented with asthma and reflects insufficient control of underlying asthma.^2^ In this study, serum periostin levels in the asthmatic children with both positive exercise and mannitol BPT were significantly greater than those in the asthmatic children with both negative exercise and mannitol BPT. Although there was no statistically significant difference, there was more moderate, persistent asthma in asthmatic children with both positive exercise and mannitol BPT than the other groups. However, it is unclear in our study whether periostin is associated only with EIB or with asthma control because we did not have a group of patients exhibiting frequent exacerbations of asthmatic symptoms. A prognostic relationship between periostin and risk of asthma exacerbations has been observed in clinical studies.^29^ There have been several studies reporting that periostin was associated with poor asthma control. In the omalizumab EXTRA study, in which subjects were required to have experienced at least one exacerbation in the previous year, severe exacerbation rates over 48 weeks in the placebo arm were 0.93 and 0.72, respectively, in the periostin-high and periostin-low subgroups.^30^ The severe exacerbation rate per year in the LUTE and VERSE lebrikizumab studies was also higher in placebo-treated patients with high serum periostin levels than in periostin-low patients (1.01 vs. 0.48, respectively).^31^

There were several limitations to the present study. First, the sample size was small. Second, we could not discuss how our findings may be linked to poor asthma control, because we did not have a group of patients exhibiting frequent exacerbation of asthmatic symptoms. Third, periostin may not be a dependable biomarker in growing children because it is an extracellular matrix protein secreted by osteoblasts. However, the levels in our study subjects aged 6–15 years old were no higher than published values for adults^26^, ^27^ and were not significantly associated with age. As few data on periostin levels in infants and children are available, such values should be investigated further in both asthmatics and healthy controls.

To the best of our knowledge, this is the first controlled observational study of the relationship between serum levels of periostin and EIB in asthmatic children. In addition, we also assessed AHR by performing both exercise and mannitol challenge tests.

## Conclusions

Serum levels of periostin were significantly greater in asthmatic children with both positive exercise and positive mannitol BPT than in those with both negative exercise and negative mannitol BPT and controls. Therefore, periostin levels may serve as a clinically useful biomarker for identifying EIB in asthmatic children.

## Declarations

### Ethics approval and consent to participate

All procedures were approved by the Medical Ethics Committee of Hallym University Kangdong Sacred Heart Hospital, Seoul, Korea, and all subjects and/or parents gave written informed consent.(IRB No. 2015-07-004).

### Consent for publication

Not applicable.

### Availability of data and materials

The datasets used and/or analyzed during the current study are available from the corresponding author on reasonable request.

### Funding

This research was supported by Hallym University Kangdong Sacred Heart hospital Research Fund (grant number: 2016-09).

## Competing interests

The authors declare that they have no competing interests.

## Authors' contributions

Ju Hwan Cho, PhD: conception and design of the study, collection of the data and analysis and interpretation of the data, and preparation and revision of the manuscript.

Kyubo Kim, MD, PhD: collection of the data, interpretation of the data and preparation of the manuscript.

Jung Won Yoon, MD: design of the study, interpretation of the data and preparation of the manuscript.

Sun Hee Choi, MD, PhD: conception and design of the study, interpretation of the data, and preparation of the manuscript.

Youn Ho Sheen, MD, PhD: conception and design of the study, interpretation of the data, and preparation of the manuscript.

ManYong Han, MD, PhD: conception and design of the study, interpretation of the data, and preparation of the manuscript.

Junya Ono, MS: collection of the data, and analysis and interpretation of the data.

Kenji Izuhara, MD, PhD: conception and design of the study, interpretation of the data, and preparation of the manuscript.

Hey-Sung Baek, MD, PhD: conception and design of the study, collection of the data and analysis and interpretation of the data, and preparation and revision of the manuscript.

All authors read and approved the final manuscript.
